# Treating without Seeing: Pain Management Practice in a Thai Context

**DOI:** 10.1155/2016/9580626

**Published:** 2016-12-01

**Authors:** Manaporn Chatchumni, Ampaporn Namvongprom, Henrik Eriksson, Monir Mazaheri

**Affiliations:** ^1^School of Nursing, Rangsit University, Pathum Thani, Thailand; ^2^Department of Nursing and Care, The Swedish Red Cross University College, Stockholm, Sweden

## Abstract

Pain management is a core nursing function, and it plays a key role in postoperative care. It is important to understand the cultural context of nursing practices and how this affects effective pain management. The aim of this study was to describe the professional and cultural framework within which pain management is practiced on a Thai surgical ward. Spradley's ethnographic methodology was used. Data were collected through 98.5 hours of field observations and interviews at a surgical ward in Thailand. Three themes were constructed that describe the way Thai nurses practiced pain management: (i) complex communications system to address pain and to respond to it, (ii) the essence of Thai-ness, and (iii) a passive approach to pain management. The results indicate that, in the response to discomfort and pain, better pain management will result if there is a shift from functional to patient-centered care. The nursing culture needs to be further researched and discussed, in order to set priorities in line with the goals of national and international organizations for improving postoperative care and promoting patient comfort.

## 1. Background

Pain management is one of the most central nursing functions worldwide. No matter what their field of expertise or function, nurses are expected to ease and relieve pain. Postsurgical pain management is always a necessity, and increasing evidence points to the conclusion that successful pain management leads to faster recovery and shorter hospitalization times. But the delivery of effective pain management is a complex process that involves not only pharmacological considerations, but also cultural ones for patients and nurses.

According to Maneewat [1], in Thailand, pain management is routine, almost ritualized, and reflects a fixed assumption of the nursing culture about the way care should be delivered. “Culture” refers to behavioral and attitudinal norms in addition to systems of meaning [2]. Culture shapes beliefs and behaviors around illness, health care practices, help-seeking activities, and receptivity to medical interventions [2–4].

Benner [5] posits that the skill of pain management is acquired and developed and is a main clinical problem-solving skill that relies on the intuition and expertise of nurses. However, nurses often provide only routine care and their practices are often more task-oriented than related to problem-solving [5].

The quality of surgical nursing care as it relates to pain management is undergoing rapid change. Nurses use empirical knowledge related to pain management strategies and interventions when managing their patients' pain [6]. Understanding the impact of the nurse-patient relationship within the nursing culture on effective pain management is a key issue in improving and optimizing pain management [1, 7–10]. The ethnonursing method has been in development since the mid-1940s and can help nurses to better understand the effects of differences in cultural beliefs, care structures, and other extramedical factors as well as the presence and effects of biases about gender, religion, and national origin [11–13].

While the nurse's role has been the focus of research on their relationship with the patient in general, there is a lack of knowledge about the influence of individual nurses on pain management [14–16] in particular. This kind of knowledge is necessary in order to improve the effectiveness of pain management practices.

This study describes the pain management practices in a Thai surgical ward context by examining the complexity of contextual meaning and the operation of the nursing system.

## 2. Materials and Methods

This study received ethics approval from the appropriate organizations, in Thailand (Code: 16/2555), and the Ethical Review of Research Involving Humans, Sweden (Code: 2012/383). The rights of the participating nurses were safeguarded through confidentiality and written informed consent. The nurses gave their consent prior to the observations and were assured of confidentiality at all levels of the study.

**Figure 1:**
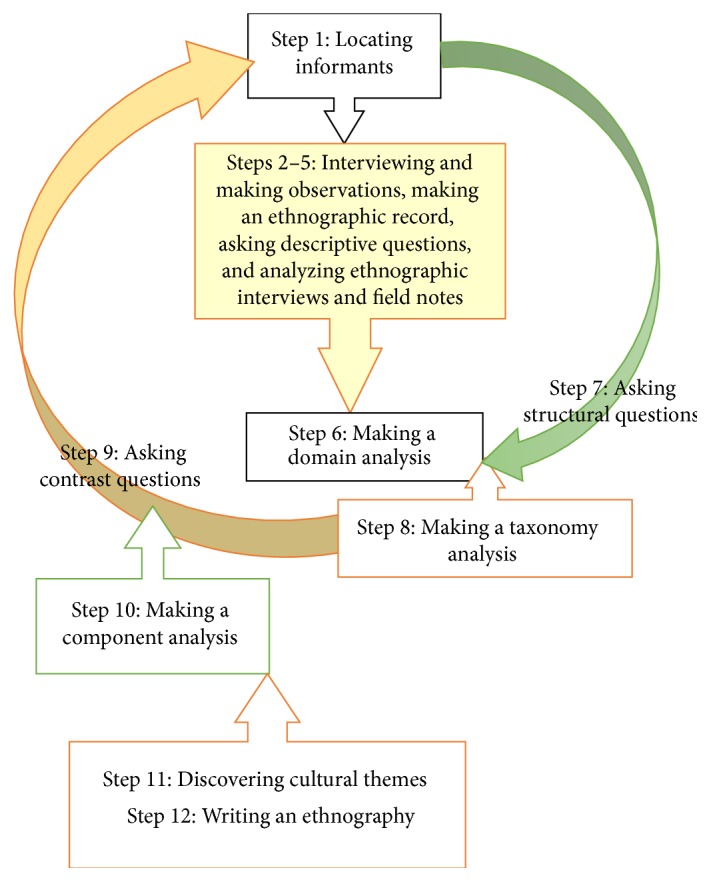
The 12 steps of the research process according to Spradley [17].

Spradley's 12-step ethnographic methodology [17] was chosen to study pain management practices and nursing care in pain management by examining how the nurses interact with their patients and other professionals in the surgical ward. This method offers a systematic approach to observing patterns within a given culture by following a prescribed research process, as illustrated in Figure 1.

### 2.1. Step  1: Locating Informants

**Figure 2:**
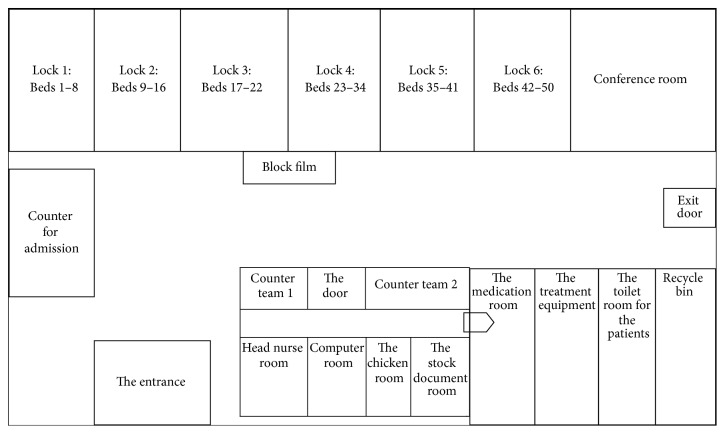
Illustration of the layout of the surgical ward.

The study took place at a 600-bed public tertiary care hospital in Bangkok, Thailand. It was conducted in the adult men's surgical ward, which provides a maximum of 50 beds (see Figure 2), and examined the nursing care provided to the patients. This surgical ward provides all types of surgeries for the male patients who are hospitalized.

A total of 40 nursing staff worked at the ward, including one head nurse (female), one subhead nurse (female), and 24 registered nurses (RNs) (20 females and 4 males). All of the nurses ranged in age from 21 to 49 years (median age: 36.5 years) and in nursing experience from 1 to 28 years with a mean average of 11.33 years. The 14 nurse's aides (NAs) and the three practical nurses (PNs) (all female) ranged in age from 32 to 54 years (median age: 38.5 years) and in working experience from 1 to 25 years with a mean average of 20.7 years. This ward had a nurse manager and an assistant manager, as well as two teams of RNs responsible for 22 to 28 beds each, with four RNs per team on the day shift, three RNs per team on the evening shift, and two RNs per team on the night shift. These teams provided care to similarly mixed surgical populations.

Since 2011, the national guidelines for pain assessment and pain management have been provided by the Royal College of Anesthesiologists of Thailand and the Thai Association for the Study of Pain. They have added to the policies of all hospitals the recommendation that healthcare providers should assess pain as the fifth vital sign. In practice, the nurses record a patient's level of pain every 4 hours (6 times per day) in a variety of methods (i.e., a verbal scale such as the Numeric Rating Scale (NRS), a graphic sheet record, and medical records) with all of the patients in the studied surgical ward [18].

Formally, the Thai nurses' curriculum involves a foundation course to educate nurses in the appropriate nursing approaches to pain assessment and pain management of their patients during the postoperative pain and recovery phases, including the recommended pharmacological and nonpharmacological interventions. In Thailand, qualified nurses must complete four years of training and are qualified at degree level, whereas PNs complete one year of nursing education to obtain a diploma, and NAs complete six months of training to obtain a certificate. Those NAs and PNs who have completed the training program relating to the monitoring of their patients' pain intensity during the postoperative period assess pain routinely as the fifth vital sign and are expected to follow a systematic recording and reporting process.

In relation to the pain protocol of the study hospital, the NAs and PNs are responsible for recording and reporting most vital signs, and when their patients' pain intensity is scored at more than five points (out of ten), they must report this to the nurses. The nurses, who are responsible for assessing pain and providing justification for administering pain relief, must then ask physicians to prescribe pain-relieving medication based on the score.

### 2.2. Steps 2–5: Collecting Data

The ethnographic fieldwork began after obtaining informed consent from the hospital administration and participating nurses. The data collection was conducted by the first author (Manaporn Chatchumni) who is a native Thai and familiar with Thai surgical patient care in general and to the study surgical ward in particular. The data collection took place over a three-month period, from July 25 to September 28, 2013, with 98.5 hours of fieldwork in total. During the fieldwork, Manaporn Chatchumni wore a formal nurses' uniform, including a name tag, and gathered data during day, evening, and night shifts, as shown in Table 1.

Both participant observations and interviews with the nurse were conducted during the fieldwork. Manaporn Chatchumni typically arrived at the surgical ward before the shifts began, as this provided an opportunity to build a rapport with the staff through informal conversation such that they were willing to be observed throughout the observation period as they addressed the patients' pain. The nurses monitored the patients' various health signs, such as checking their intravenous fluids, administering suction, and monitoring the vital signs of the patients for 10–20 minutes of every hour, as is considered routine care. Due to the ethnographic nature of the research method, participants were not chosen for interview based on specific selection criteria. Rather, they were interviewed depending on their performance of tasks relating to pain management practices during the observations. During an interview, Manaporn Chatchumni would follow the nurses for 5–10 minutes while taking notes and tape recording the interviews. She also observed the nurses' care practices and/or how they treat their patients' pain, asking questions in order to clarify their reasons for making decisions in relation to pain management. For instance, one of the interview questions was* what pain medication will you give and why do you treat him?* Manaporn Chatchumni would occasionally study and assess the recordings in the early mornings and discuss the methods of recording and summarizing the data at each step with the second author (Ampaporn Namvongprom), who also has expertise in the Thai context.

### 2.3. Steps 6–12: Analyzing Data and Writing up the Ethnography

All collected data, in terms of field notes, memos, and transcribed interviews, were analyzed by following the steps outlined in Figure 1. The first step was to review responses to the query in terms of word frequency and their semantic relationship in relation to pain management by using NVivo10 [19]. This qualitative data analysis program helped to make inquiries of the data and to sort terms and identify patterns in word frequency and by their sematic relationship to pain management. This was done to identify matches pertaining to utterances with a minimum length of 30 words, as this was influential in understanding the meaning of the interviews, memos, observations, and field notes.

Second, Manaporn Chatchumni read all of the text from the data and captured text line by line, focusing on the consistency of the meaning of culture in relation to pain management. She then categorized the results in the appropriately classified subgroups.

Third, Manaporn Chatchumni read the data within each of the subgroups (terms with the same meaning unit) in order to logically represent them in broader domains. The final step included applying the terms of analysis through a circular process. Domains were divided into three groups by establishing semantic relationships. The data were then sorted in Microsoft Excel 2010 which was used to organize them into three domains (including 226 terms in Thai language).

To identify additional terms and constantly reflect upon the categorization that should be included in each domain, structural questions were posed for the purpose of discussion among the first, third, and last authors (Manaporn Chatchumni, Henrik Eriksson, and Monir Mazaheri, resp.). For example, “what is Thai culture, especially with regard to what was happening in pain management practices?” The verification questions were also considered as a type of structural questioning and these gave credibility to each domain (e.g., “what is the Thai nurses' approach to pain management practices, and how it is distinctly the general approach?”)

After extensive discussions among the authors, certain domains were chosen for further analysis, as they corresponded well with the aim of the study. The next step involved an analysis of the terms associated with the selected domains, which were organized into taxonomies. Some of the terms related to familiar meanings were classified into first, second, and third level categories and were hierarchically arranged into a complete taxonomy, as is typically done in analyzing semantic relationships. This process was used throughout the study of each of the taxonomies.

In order to discover the differences among each of the categories, subcategories, and included terms, follow-up questions were posed and NVivo10 [19] was employed to search through the data for any additional terms and different structural questions were also posed. The purpose of asking contrasting questions was to narrow the nurses' perspectives based on the differences that existed among the terms in each domain of their pain management.

As the process continued, it became obvious that there were several interconnected attributes within each domain. The component analysis that followed (see Figure 1) was dedicated to the overall validation of the complexity of the dimensions of postoperative pain management in nursing practices that had been formed during the process. This analysis was constructed to interconnect with various ways of communicating, including parallel terms related to this dimension. Triangulation of the data was achieved in analyzing all of the information, including an element of the data sorted by NVivo 10 [19]. Furthermore, the analysis was supported by the principles of trustworthiness by promoting critical reflection and sharing between all of the authors and by taking into consideration their own experiences and perceptions within their discussions. In order to further promote the trustworthiness of the results, the descriptions within this summary have included an abstract analysis of all domains and subgroups to provide an additional interpretation of the data as a series of understandings in order to help to answer the overall aim of the study.

The final step was to write the ethnography, placing the most important themes in context as well as presenting the cultural themes that were revealed by the research process.

## 3. Findings and Discussion

The pain management practices in the studied Thai surgical ward are best described and discussed within the following three themes: (i) complex communication system to address pain and to respond to it, (ii) the essence of Thai-ness, and (iii) a passive approach to pain management.

This study has important implications for the practice of pain management in Thailand because it reveals structures where nurses are, metaphorically, “treating without seeing” when seeking to relieve patients' pain.

### 3.1. Complex Communication System to Address Pain and to Respond to It

**Figure 3:**
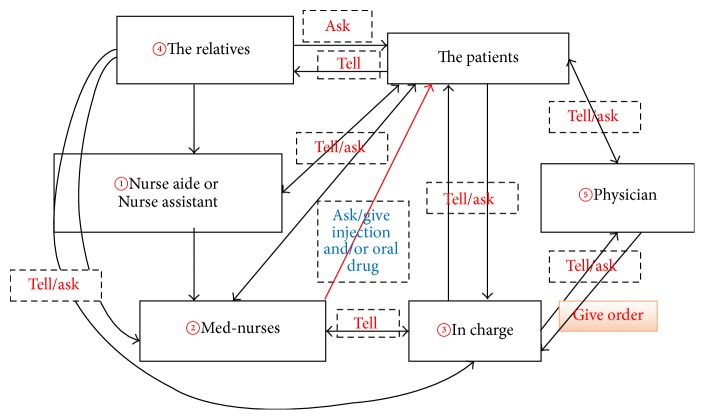
Illustration of the complex flow of communication.

The pain management practices in the Thai surgical ward involved a complex communications network that included the direct participants (nurses, patients, and physicians) and intermediaries (relatives and nursing aides). Nurse-patient communication was most often not a direct communication between patient and nurse but rather was a message relayed by an intermediary. This approach required interactions among all the players and the participating intermediaries, who might be one or many in number (see Figure 3). The complex and multiperson communication system to address pain and to respond to it (see Figure 3), together with distinctive aspects of Thai culture, led to a relatively passive approach to pain management and shaped the pain management practices of the Thai nurses we observed.

Sometimes pain was documented and actions were taken to alleviate it without there ever being direct communication between the patient and the nurse. For instance, a relative reports pain levels to an NA, who then reports to a nurse, who then reports to the in-charge nurse, who finally discusses the matter with the physician. The NA/PN can act as the “intermediate messenger,” conveying information to the med-nurses and in-charge nurses, who can then dispense medication after contacting the physician, who prescribes pain medication. Having multiple people and channels of communication in the treatment process increases the chances of delayed response to patients in pain as well as miscommunication.

Relatives acted as intermediaries in communicating the pain message, even though the nursing staff for each shift collectively assessed pain levels along with vital signs of their patients every four hours, consistent with national guidelines. During an assessment, the nurses paid particular attention to nonverbal communication by processing patients' nonverbal responses and their bodily expressions such as certain visible signs of pain (e.g., sweating) and/or body language (e.g., lying still, keeping very tense). The following is an excerpt from the field notes, in which the nurse responded to the patient's discomfort and pain:

The med-nurse was responsible to the physician and was required to focus on the patient's treatment in the way of routine care, such as wound dressing, antibiotic injections, suction, feeding, and the monitoring of intravenous fluids, while the patients would communicate with them as to their needs with regard to medication for reducing pain./…/by asking about their pain level; “Do you have pain?” and “How much pain do you feel?”/…/the patient would reply with a pain scores of >5 out of 10 points./…/then/…/to provide preliminary medication in order to relieve the patient's pain./…/reported on an evaluation of the patient's pain to the in-charge nurse by remarking on the pain record form and the nurses' notes. (Field notes, @ 3 pm. July, 2013)

MC: Why did you do that?

Eumh!! That is 4 or 5 out of 10 points. Here, I'm assessing the level of the patient's pain by asking,/…/*“Do you have pain? Somewhere?”* If he can eat something because he does not have any conditions after surgery, I then decided to give him paracetamol. But, if he cannot eat following the physician's orders, it is possible to advise him to do deep breathing and to raise or lower the position of the head on the pillow. (Interviews K3)

The mutual communication between the patient as the main recipient of the treatment and the nurse as providing the nursing care in regard to pain was mediated by NAs or PNs, who would assess and record the pain score. Ineffective communication (communication errors) occurred primarily at two points: through the patients' descriptions and the observation of the patients' level of pain by the staff.

The paper documentation of pain levels was not routinely used as a basis for prescribing pain medication or administrating other pain relief strategies. The physicians obtained information about pain and its characteristics through their direct contact with patients or through the in-charge nurses. The nurse-physician communication also had intermediaries and messages were transferred by the head nurse/nurse in charge to the physician, and vice versa.

After the administering of pain medication, the majority of the patients conveyed their sense of satisfaction or discomfort through relatives. The relatives were usually able to stay with the patient. The primary purpose of this was so that the patients could receive a sense of comfort from having their relatives at their bedsides and to instill a sense of trust in the institution.

Previous studies have recognized that the nursing unit culture might influence the practice of pain management [1, 20]. However, the practices differed in each unit, according to the individual approaches of the nurses who responded to the patient's expressions of pain and discomfort. This could be perceived as the norm and as the standard of the nurses' practical knowledge. Not only the unit culture but the care recipient culture should be considered in assessment as well as nursing strategies for responding to discomfort.

Considering the theme of “the essence of Thai-ness” illustrated the influence of the essence of nursing culture within this practice. Our study shows the importance of being aware of patients' ways of responding to pain and discomfort within “the essence of Thai-ness” in order to promptly and accurately assess the condition of patients. Not complaining of pain and discomfort was not equal to not* having* pain or discomfort among Thai patients. Therefore, nurses need to use other strategies to recognize pain and ensure patients' comfort.

For pain management, its documentation must comply with the national guidelines developed by the Royal College of Anesthesiologists of Thailand and the Thai Association for the Study of Pain [20] for the management of chronic pain and acute pain, and it has been ordered that these guidelines are implemented across all wards in Thailand. Despite this goal, our study shows that the implementation of the national guidelines has not been fully achieved.

With the publication of the guidelines, pain is now considered to be the fifth vital sign and must be assessed every four hours for postoperative patients in surgical wards in Thailand. Our study, however, showed that the data nurses accumulated in relation to the levels, quality, and characteristics of patients' pain did not reflect evidence-based practice. Moreover, the indirect, multiperson system of communication diminished the accuracy of the collected evidence [1, 21]. The paper documentation of the collected data took considerable time, yet it did not function as the basis for making decisions relating to pain alleviation strategies by physicians or even the nurses themselves.

The issue of poor implementation of guidelines and protocols might be an effect of Thai culture which, as a result, leads to no change occurring in practice, which is a well-known problem in health care. Health policymakers need to further evaluate the implementation of the guidelines and make the necessary adjustments to optimize pain management practices, minimize patient discomfort, and align Thai practices with current international best practices.

### 3.2. The Essence of Thai-Ness

The informants believed that it is their duty and responsibility to effectively apply pain management practices and they described how they responded to the discomfort and pain of the patients. They saw nursing as an intentional act in which nurses are willing to help their patients and alleviate the patients' discomfort and postoperative pain. This was evident when they helped manage their patients' pain (e.g., rapidly responding to the patients' discomfort and pain with treatment, including giving an analgesic injection).

It was observed that an individual's “cultural arena” (the history of traditional aspects) is important. For instance, the majority of the informants understood the impact of Buddhist traditions when providing care to their patients, which involved* merit making *as well as the expectation of managing pain.

There is also the cultural aspect of the language used on the ward, reflected in particular expressions such as* mee nam jai, *which means “offering to help others,” rather than “asking for help” within the nursing team. The informants referred to teamwork, communication, and collaboration skills, all of which are considered to be aspects of Thai-ness, as well as an expression of kindness and a desire to help other people. In communication, this* mee nam jai *feature acknowledges the relationships that promote the development of a functional ward.

Being subtle and indirect are valued characteristics of Thai culture that we observed to be practiced in lieu of clear and direct communication regarding pain. The nurses said that some patients might not complain about their pain and discomfort, which can be an obstacle to the nurses' correct assessment of their patients' levels of comfort.

One aspect of this is seen in the use of the term* kreng jai* in Thai culture, which has been characterized as “the essence of Thai-ness.” This is conveyed in nursing care, and though precisely what* kreng jai* entails can be hard to describe, it is a concern for the patients who feel uncertain or become distanced from the nurses, as demonstrated in the quotation below.

There are several ways to perceive a patient's pain. Sometimes, their relatives can tell us about their pain./…/but I may walk directly to the patient's bedside. He may look at me with an unspoken expression of* kreng jai*. His face may convey an expression of pain,/…/, he may tell me* mai pen rai thon dai *(meaning “no problem, I can endure it”). However, it does not actually correspond with the expression of his face, which is expressing pain./…/. (Interviews K2)

A similar cultural practice was observed among nurses in the surgical ward. The nurses believed in helping the other nurses without waiting for a request. In fact, not requesting help was often beneficial in terms of receiving more assistance.

These findings revealed the presence of a personal barrier in relieving pain that needs to be seriously considered by nurses when pursuing pain management. Personal beliefs and life philosophies have direct consequences for the way patients seek care or express themselves, as well as the way in which nurses communicate with, hear, and respond to their patients. Thai nurses should offer help to patients more often than they currently do, and communication should be more direct. Nurses should speak with their patients, reducing or eliminating the role of relatives as intermediaries. This would be a natural consequence of* mee nam jai* and would improve nurses' ability to rapidly respond to patients' pain [8].

### 3.3. Passive Approach to Pain Management

Nurses were observed when responding to their patients' pain, and their approach was perceived as mostly a formal practice in the paper documentation, including medical records, nurse's notes, and physicians' progress notes. Regular pain assessment (every four hours) was followed by charting pain levels, both on bedside forms and in patients' journals. Completing the paper documentation occupied a considerable amount of nurses' time on every shift.

Although the pain assessment involved routine monitoring every four hours, the nurses often waited for the patients to ask for analgesics. Patients, in turn, were likely waiting to be offered help. The nurses' passive approach to alleviating patients' pain was described in the field notes. For example, we have the following field notes:

The relative of the patient approached the nurses' counter and stated the following directive to the nurse's aide (NA):* “You have to give drugs to the patient in bed 38 to reduce their pain.”*

The NA then conveyed this message to med-nurse team 2:* “(Name) in bed 38 needs drugs.”*

Med-nurse team 2 would recheck the patient's chart and walk into the medication room to preparing a morphine (MO) injection of 3 mg for the patient.

While the med-nurse was giving the MO injection to the patient, he would then ask him to record his level of pain by saying:* “Please indicate the level of pain you feel by recording the appropriate score.”*

The patient replied:* “10 points.”*

The med-nurse then said:* “I will give you medication to reduce your pain.”* (Field notes @ 4.23 am. August, 2013)

The nursing staff usually gave paracetamol as a basic analgesia strategy. The nurses are aware of and frequently cited significant side effects of opioids (e.g., morphine, pethidine), such as respiratory depression. However, there were no obvious routines to evaluate the impact of painkillers. In several situations the effects of the medication given to the patients were only evaluated if the administration was intravenous and following a physician's orders.

The nurses also used nonpharmacological interventions, such as changing the position of the patient's body and guiding the patient through deep-breathing activities, as revealed in the interviews.

Mostly, I was asking the patient “How are you?” or “Are you feeling pain?” and it was revealed that around one to three patients were enduring pain. Also, the patient might just say/…/a small amount of pain after they have changed their position and/or altered their breathing. In certain cases, a patient may be experiencing something like some colicky pain and they said/…/an estimated two to three points out of ten. (Interviews K2)

The med-nurse was talking with the patient to try to get them to change their body position and to slow their breathing through measured inhalation and exhalation. She told them that if they were still feeling pain, then they should let the med-nurse know. (Field notes @ 7:55 pm, August, 2013)

Thai people consider the influences of local culture in understanding the day-to-day pain management practices of nursing, the reality of workplace management, and the strategies in place for the patients' postoperative pain management in the surgical ward. The differing cultures found within different wards in existing healthcare organizations have also been shown to have a significant influence on the nursing culture and how the organizational culture influences nurses' thinking and decisions [13]. Professional ambitions for sustainable improvement in postoperative care related to pain management, such as those of the Joint Commission for Accreditation of Healthcare Organizations (JCAHO) [22], are valuable resources for health care organizations when changing their pain assessment and management processes to meet the new standards [23, 24].

Because nurses are the primary caregivers in pain management situations, they should be involved in the development of protocols for postoperative pain management based on adequate assessment and immediate action [9, 10, 21].

### 3.4. Strengths and Limitations

One limitation of the study was that the data were not collected directly from patients. However, important data were gathered through observation of what actually took place on the ward. The first author (Manaporn Chatchumni) was a novice researcher in terms of using the ethnographic method, but the research team had several members including those with expertise in research, nursing, and the Thai context. Some steps of the verbatim translations were analyzed by Ampaporn Namvongprom, who has expertise in qualitative research and is a native Thai speaker, in order to clarify any vagueness in the language. Spradley's rigorous ethnographic method was used to examine the specific approaches used to execute the pain management strategy, while considering that the nursing practice is shaped by the specific Thai context in which it occurs. The method was used to study the cultural appropriateness, as well as the process of the data collection that was carried out in accordance with Spradley's model [17, 25].

## 4. Conclusions

This study may have important implications for the practice of pain management in Thailand, as it reveals structures where nurses are metaphorically “treating without seeing” when aiming to relieve patients' pain. The study shows that the overall nursing organization should be developed in line with professional ambitions to improve postoperative care as it relates to pain management and that nurses should be setting priorities accordingly.

Achieving improvements in postoperative pain relief in Thailand requires a strategic shift, from functional to patient-centered care. Rather than waiting until patients (or their relatives) request pain medication, nurses should directly and continuously monitor patient discomfort and pain levels in order to respond promptly to increased pain.

Efforts must also be made to create and sustain a direct communications path from patient to nurse, thus eliminating delays and misinterpretations and maximizing the opportunity to promptly relieve pain. Thai nurses need to assume a larger and more active role in the postsurgical pain control process.

## Figures and Tables

**Table 1:** Data collection (duration).

Field work (observation)	Amount of observation
During day shift	24.5 hours
During the change from day shift to evening shift	42 hours
During the change from evening shift to night shift	32 hours
*Total*	*98.5 hours*

Resulting data	94 double-spaced pages of observation notes
